# Inhibiting mycobacterial tryptophan synthase by targeting the inter-subunit interface

**DOI:** 10.1038/s41598-017-09642-y

**Published:** 2017-08-25

**Authors:** Katherine A. Abrahams, Jonathan A. G. Cox, Klaus Fütterer, Joaquín Rullas, Fátima Ortega-Muro, Nicholas J. Loman, Patrick J. Moynihan, Esther Pérez-Herrán, Elena Jiménez, Jorge Esquivias, David Barros, Lluís Ballell, Carlos Alemparte, Gurdyal S. Besra

**Affiliations:** 1 Institute of Microbiology and Infection, School of Biosciences, University of Birmingham, Edgbaston, Birmingham, B15 2TT UK; 2 School of Life and Health Sciences, Aston University, Aston Triangle, Birmingham, B4 7ET UK; 3 Tres Cantos Medicines Development Campus, GlaxoSmithKline, Severo Ochoa 2, 28760 Tres Cantos, Madrid Spain

## Abstract

Drug discovery efforts against the pathogen *Mycobacterium tuberculosis* (*Mtb*) have been advanced through phenotypic screens of extensive compound libraries. Such a screen revealed sulfolane **1** and indoline-5-sulfonamides **2** and **3** as potent inhibitors of mycobacterial growth. Optimization in the sulfolane series led to compound **4**, which has proven activity in an *in vivo* murine model of *Mtb* infection. Here we identify the target and mode of inhibition of these compounds based on whole genome sequencing of spontaneous resistant mutants, which identified mutations locating to the essential α- and β-subunits of tryptophan synthase. Over-expression studies confirmed tryptophan synthase as the biological target. Biochemical techniques probed the mechanism of inhibition, revealing the mutant enzyme complex incurs a fitness cost but does not prevent inhibitor binding. Mapping of the resistance conferring mutations onto a low-resolution crystal structure of *Mtb* tryptophan synthase showed they locate to the interface between the α- and β-subunits. The discovery of anti-tubercular agents inhibiting tryptophan synthase highlights the therapeutic potential of this enzyme and draws attention to the prospect of other amino acid biosynthetic pathways as future *Mtb* drug targets.

## Introduction

*Mycobacterium tuberculosis* (*Mtb*), the causative agent of the pulmonary disease, tuberculosis (TB), is considered to be the world’s most successful pathogen^1^. According to a WHO survey in 2015^2^, *Mtb* was shown to be responsible for 10.4 million new cases of TB, which included 480,000 new cases of multi-drug resistant (MDR)-TB, with an estimated 1.8 million deaths and 400,000 deaths resulting from co-infection with HIV. In 2015, the United Nations adopted the objective to end the global TB epidemic by 2030^2^. To achieve this goal, there is an urgent requirement to develop new diagnostics, vaccines and treatment regimens.

To limit the potential for resistance, TB therapy has, for decades, used a 4-drug cocktail consisting of isoniazid (INH), rifampicin (RIF), pyrazinamide and ethambutol, which inhibit a diverse set of essential metabolic nodes^3–6^. Nonetheless, strains with dual resistance to INH and RIF (classed as multi-drug resistant), and more extensively against fluoroquinolones and injectable second-line drugs, are on the rise. Clearly, an effective strategy to counter resistance has to include not just novel compounds, but perhaps more importantly targets that have not yet been subjected to selective pressure by antibiotics.

**Figure 1:**
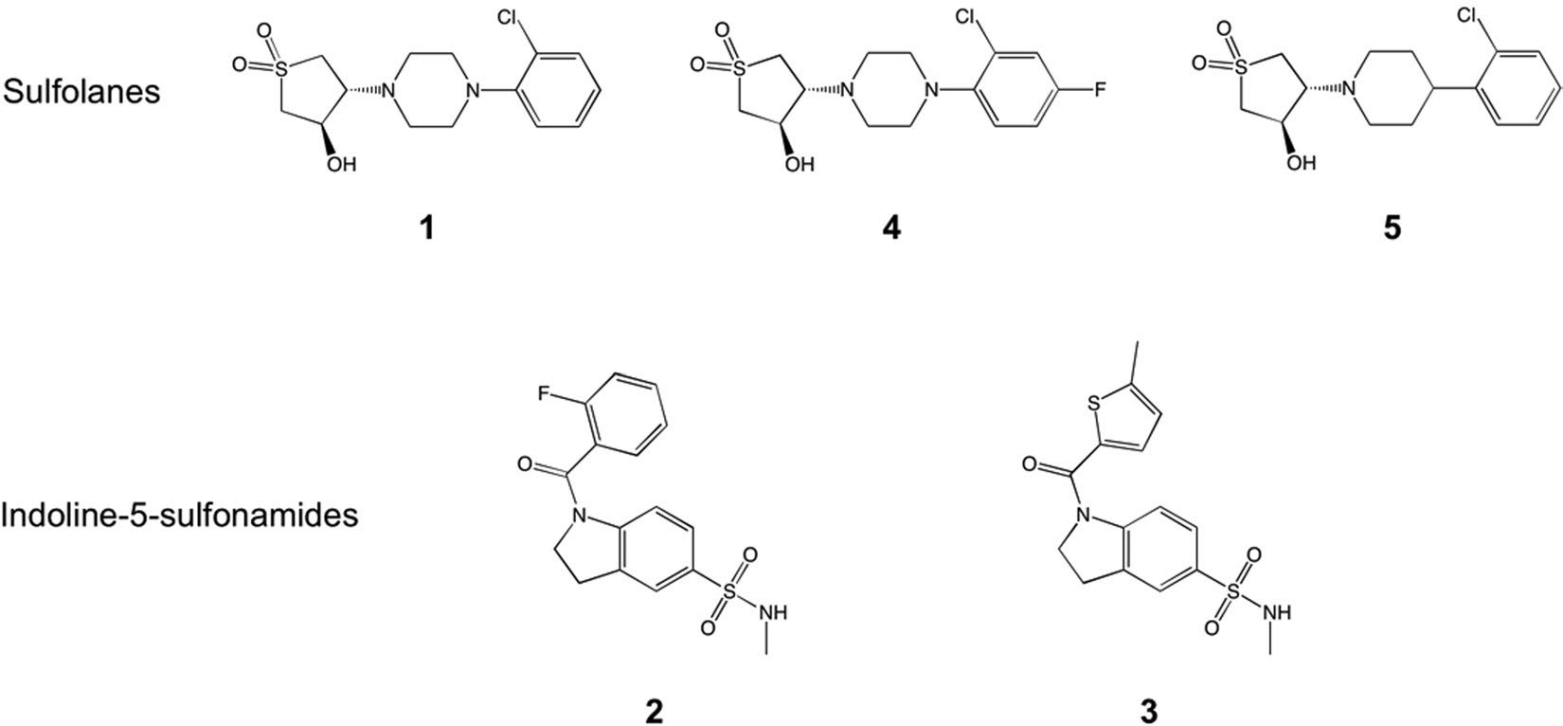
Structures of compounds **1**–**5**.

In recent years, TB drug discovery has been dominated by whole cell phenotypic high throughput screening (HTS) campaigns of extensive compound libraries against *Mtb* ^7, 8^. In 2013, GlaxoSmithKline (GSK) published a collection of 177 non-cytotoxic compounds, known as the ‘TB box set’, with activity against *Mtb* H37Rv^7^. This set has been extended to include a total of 227 compounds^8^ and has led to a broad effort of target assignment. However, to progress these hit compounds to leads and clinical candidates, target validation is crucial, enabling optimization of chemical scaffolds by medicinal chemistry efforts. Target assignment also offers the potential to discover novel drug targets, which can be exploited in inhibitor design. In this work, we have recognized the potential of an exciting novel anti-tubercular drug target, tryptophan synthase, which catalyzes the final step in tryptophan biosynthesis. Tryptophan is classified as an essential amino acid in humans and must be acquired through the diet. This requirement alleviates the concern of common targets within humans. Here, we describe the target identification and hit optimization for compounds **1**, **2** and **3** (Fig. 1), and have characterized their interaction with the target tryptophan synthase. Finally, the target assignment of the tryptophan synthase highlights the plausibility of amino acid biosynthesis pathways as suitable and underexploited *Mtb* drug targets.

## Results

### Identification of sulfolanes and indoline-5-sulfonamides as anti-TB hits

GSK continuously screens the new chemical diversity in its compound collection in search for new hits with anti-tubercular potential. As part of these efforts, we have recently identified two new chemical scaffolds with *in vitro* activity against *Mtb*: sulfolanes, represented by compound **1** and indoline-5-sulfonamides, represented by compounds **2** and **3** (Fig. 1). The *in vitro* profile of these compounds is shown in Table 1. All three compounds have good potencies against *Mtb*. Importantly, the compounds do not show cytotoxicity against HepG2 cells up to 50–100 µM. Lipophilicity is in the desired range (cLogP varies between 1.63 and 2.05) while Property Forecast Index (PFI), that has shown a great potential to predict drug developability^9^, is slightly high for compound **3**. This fact, together with the considerable planarity of the molecule, may explain its poor solubility. The other representative of the indoline-5-sulfonamide series (**2**) exhibits enhanced solubility but low stability in the presence of not only mouse but also human microsomal fraction. Disappointingly, medicinal chemistry optimization and structure-activity relationship (SAR) explored around the initial hits (compounds **2** and **3**) was found to be very limited and after a significant number of analogues had been synthesized and evaluated, the series was abandoned.

**Table 1:** *In vitro* profile of compounds **1**–**5**.

Compound	1	2	3	4	5
*Mtb* H37Rv MIC (µM)^a^	0.76	5.6	1.1	2.25	0.5
HepG2 Tox50 (µM)	>50	>100	>100	>100	>100
ClogP	2.05	1.63	1.69	2.66	2.51
PFI^b^	4.53	5.80	6.28	5.43	5.22
Cli (mL/min·g) mouse	15.7	39.5	27.1	<0.5	73.7
Cli (mL/min·g) human	0.92	15.7	1.9	1.1	1.7
CLND Solubility (µM)	>464	140	19	434	397
AMP Permeability (nm/s)^c^	380	260	427	625	520

^a^The anti-tubercular activity against *Mtb* H37Rv was performed as previously described^35^. ^b^Property forecast index (PFI) is defined as the sum of chromatographic logD at pH = 7.4 + number of aromatic rings. ^c^Artificial membrane permeability (AMP) was determined following published protocols^36^.

The overall profile of the sulfolane **1** is very encouraging. Additional chemical space around the active compounds in the sulfolane series, allowed further optimization, which is exemplified by compounds **4** and **5**. For instance, compound **4** presents a reasonable compound with *in vitro* activity and high metabolic stability, and it is therefore suitable for oral *in vivo* efficacy studies. We also decided to progress compound **5**, due to its enhanced potency. However, in this case we used a subcutaneous administration to avoid first pass metabolism and mitigate the effect of its low microsomal stability. The structures and *in vitro* data of compounds **4** and **5** are shown in Fig. 1 and Table 1, respectively.

**Figure 2:**
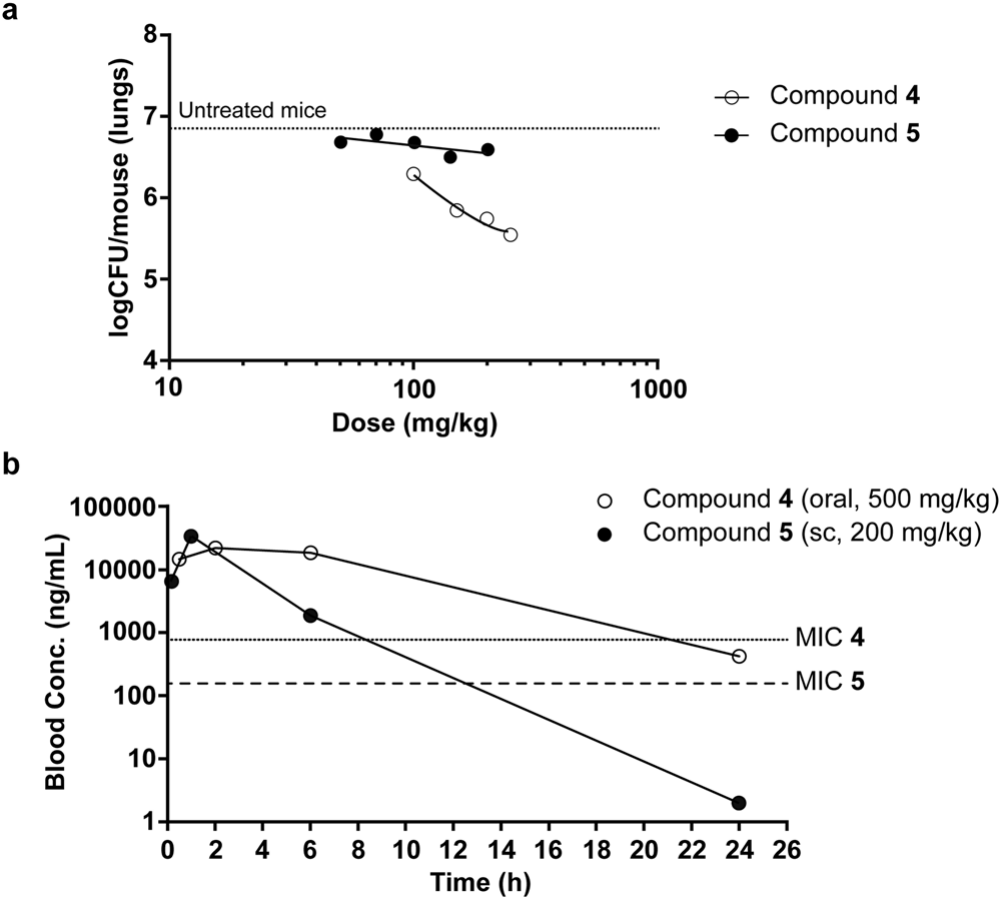
The *in vivo* anti-tubercular activity of the sulfolane series. (**a**) Therapeutic efficacy of **4** and **5** against *Mtb* H37Rv *in vivo*. C57BL/6 J mice (one mouse per dose) were infected by intratracheal instillation with 10^5^ CFU *Mtb* H37Rv per mouse. The mice were treated orally once a day from day 1 to day 8 and sacrificed on day 9. (**b**) **4** and **5** were measured in the blood of the infected mice (oral and sc, subcutaneous). Samples from the mice used in the efficacy experiment were taken and analyzed. Graph shows *Mtb* H37Rv MIC of the inhibitors as a reference.

Figure 2a shows the results obtained when the efficacy of both compounds was measured in an acute infection assay in C57BL/6 mice. Compound **4** (oral, 100–500 mg/Kg) proved to be efficacious and a dose/response curve was obtained. At the maximum dose evaluated, 350 mg/Kg (the mouse dosed at 500 mg/Kg had to be withdrawn due to adverse effects), a reduction of 1.4 log colony forming units (CFU) was observed when compared with untreated mice. In the same study and despite its higher *in vitro* potency, compound **5** did not show a statistically significant response. Blood samples were taken from the mice used in the efficacy experiment to quantify the concentration of compounds **4** and **5**. The data is depicted in Fig. 2b. Although the number of time points was limited and accurate pharmacokinetic parameters cannot be calculated, a significant maximum concentration and exposure of both compounds was evident. The reason for the lack of efficacy of compound **5** is still unknown. A major difference in distribution to the lungs between compounds **4** and **5** is not expected considering their structural similarity. The lower half-life of compound **5** could help explain its inactivity *in vivo* if the minimum inhibitory concentration (MIC) is a relevant driver for the efficacy. Further *in vivo* experiments will be needed to test this hypothesis. The anti-tubercular *in vivo* efficacy observed with compound **4** constitutes a successful hit optimization for the sulfolane series and for the value of tryptophan synthase as a therapeutic target.

### Target identification of the sulfolanes and indoline-5-sulfonamides series

Establishing the mode of action of inhibitory compounds is an essential step in drug discovery. The identification of biological targets is often pursued using a variety of omics-based techniques, with whole genome sequencing (WGS) of resistant mutants a proven successful starting point^10–12^. Accordingly, this was the preliminary method employed to elucidate the targets of compounds **1**, **2** and **3**.

The MICs of compounds **1**, **2** and **3** were established in the *Mtb* model organism, *Mycobacterium bovis* BCG and determined to be 1 μM, 5 μM and 0.7 μM, respectively. Spontaneous resistant mutants were generated at 5× and 10× MIC for each compound. The frequency of resistance (FoR) for each compound at 5× and 10× MIC, respectively, were as follows: compound **1**, 2 × 10^−8^ and 1 × 10^−8^; compound **2**, 3 × 10^−8^ and 7 × 10^−8^; compound **3**, 9 × 10^−8^ and 2 × 10^−8^. The gDNA from a number of spontaneous resistant mutants generated was extracted and subjected to WGS as detailed in Table 2. High frequency, high-quality (statistically relevant) single nucleotide polymorphisms (SNPs) were identified in the mutant gDNA compared to the sequenced wild-type reference strain (Genbank accession number NC_008769.1). SNPs located to: *ansP2*, encoding asparagine permease; *trpA*, α-subunit of tryptophan synthase; *trpB*, β-subunit of tryptophan synthase. TrpA and TrpB form tryptophan synthase, a heterotetramer of α- and β-subunits (with the configuration of αββ’α’). Asparagine permease is an asparagine transporter^13^ and tryptophan synthase catalyzes the final step in tryptophan biosynthesis.

**Table 2:** Whole genome sequencing results of spontaneous resistant mutants raised against compounds **1**, **2** and **3**.

Compound	Mutant	× MIC	Genome position of SNP	Gene	Base change	Amino acid substitution
**1**	1	5	*447564*	*ansP2*	tGg/tCg	W110S
			1817251	*trpB*	tTt/tGt	F293C
	2	5	447564	*ansP2*	tGg/tCg	W110S
			1816935	*trpB*	Ttc/Ctc	F188L
	3	10	447564	*ansP2*	tGg/tCg	W110S
			1816935	*trpB*	Ttc/Ctc	F188L
**2**	1	5	1816927	*trpB*	aAt/aGt	N185S
	2	5	1816927	*trpB*	aAt/aGt	N185S
	3	5	1816924	*trpB*	aTc/aGc	I184S
	4	5	1816924	*trpB*	aTc/aGc	I184S
	5	5	1818011	*trpA*	Gac/Aac	D136N
	6	10	1816996	*trpB*	cCg/cTg	P208L
	7	10	447564	*ansP2*	tGg/tTg	W110L
**3**	1	5	447564	*ansP2*	tGg/tTg	W110L
	2	5	447564	*ansP2*	tGg/tTg	W110L
	3	10	447564	*ansP2*	tGg/tCg	W110S
	4	10	447564	*ansP2*	tGg/tTg	W110L
	5	10	1816927	*trpB*	aAt/aGt	N185S
	6	10	1816927	*trpB*	aAt/aGt	N185S
	7	10	1816937	*trpB*	ttC/ttA	F188L

Spontaneous resistant mutants were raised in *M. bovis* BCG using the compound and MIC specified. The genome position of the mutation is stated, along with the codon change (the capital letter denotes the base change) and resulting amino acid substitution.

### Target validation

**Figure 3:**
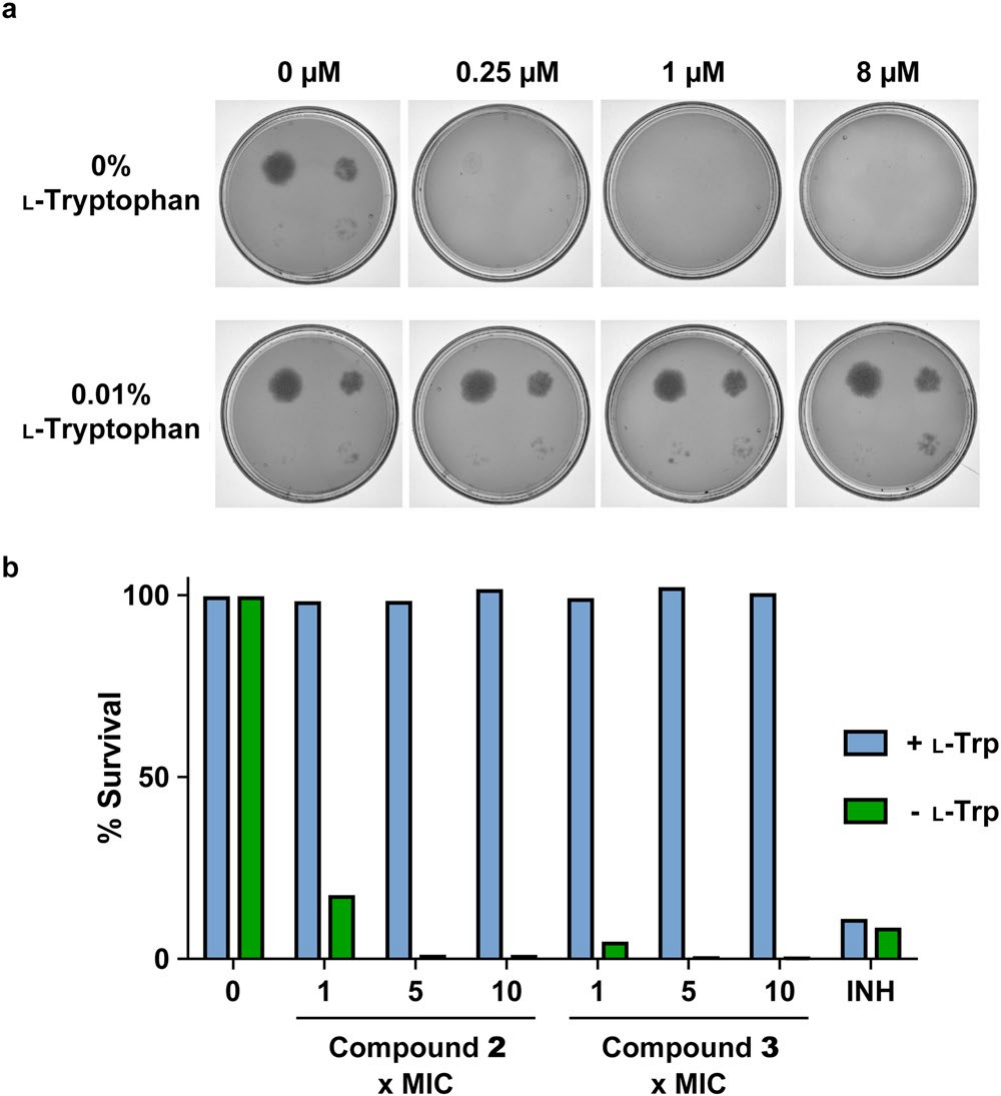
l-Tryptophan rescue of *M. bovis* BCG inhibition by compounds **1**, **2** and **3**. (**a**) The MIC of compound **1** was analyzed in the presence and absence of 0.01% l-tryptophan on solid minimal media by plating 10^4^, 10^3^, 10^2^ 10^1^ cells (clockwise from top left). (**b**) The survival of *M. bovis* BCG in liquid minimal media with 0×, 1×, 5× and 10× MIC compounds **2** and **3** was analyzed in the presence and absence of 0.01% l-tryptophan. Isoniazid (INH) was used as a control, where l-tryptophan does not rescue the inhibition caused by this cell wall biosynthesis inhibitor.

The results from the WGS indicate that the inhibitors target a process involving amino acid transport or metabolism. Therefore, to pursue this line of investigation, the MIC of wild-type (WT) *M. bovis* BCG with compound **1** was evaluated in minimal media (solid) in the presence and absence of 0.01% l-tryptophan, with glycerol as a carbon source. The results are displayed in Fig. 3a. In the absence of l-tryptophan, the MIC was <1 μM. However, in the presence of l-tryptophan, the MIC was increased to >8 μM. This indicates that l-tryptophan rescues the inhibition by compound **1**, where the availability of the amino acid in the media alleviates the necessity of l-tryptophan *de novo* synthesis. The same response was observed with compounds **2** and **3**; in liquid minimal medium, l-tryptophan rescued the inhibition by the compounds at 1×, 5× and 10× MIC as shown in Fig. 3b. In combination with the WGS results, it was highly plausible that compounds **1**, **2** and **3** target tryptophan synthase.

**Figure 4:**
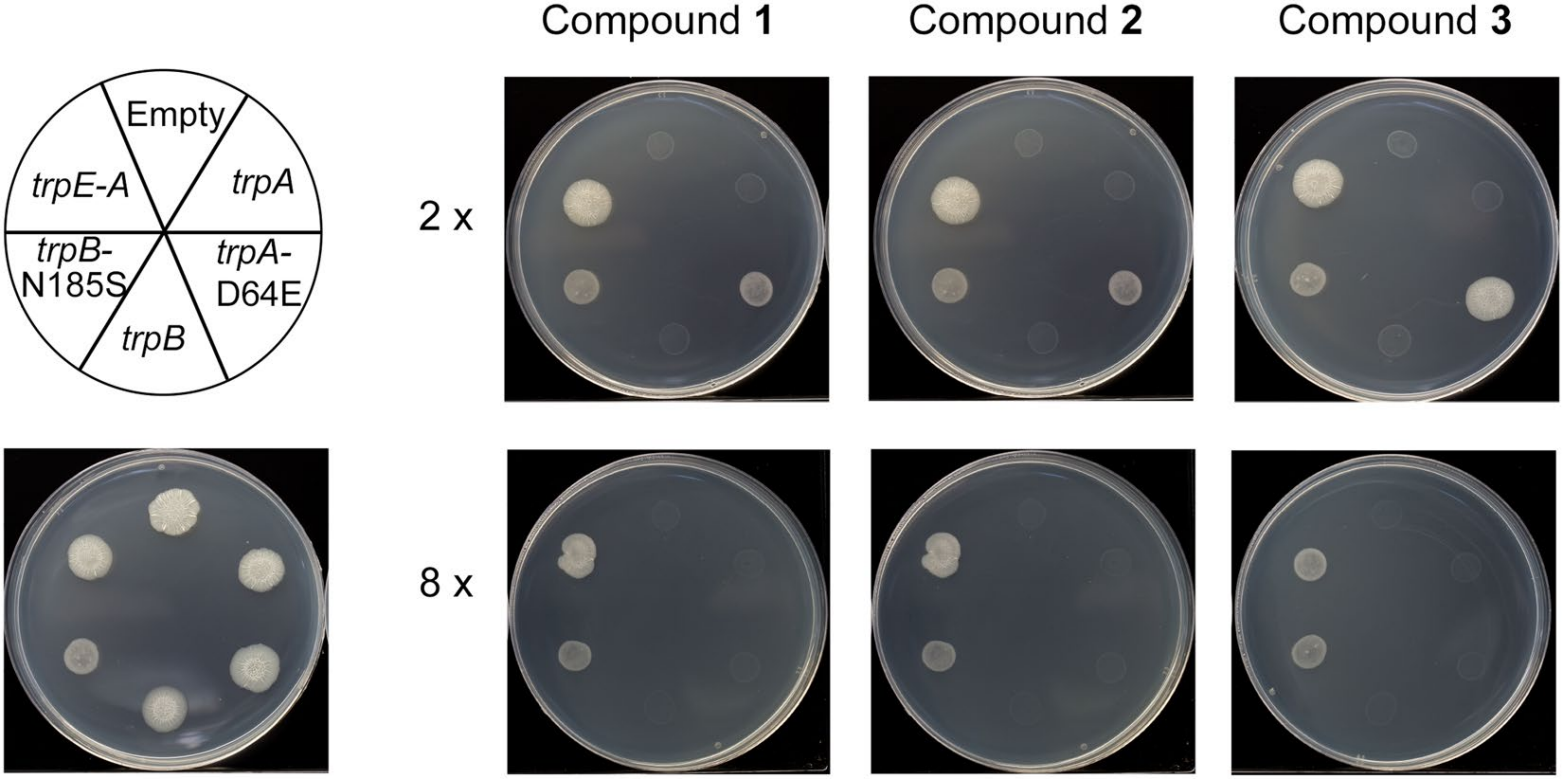
Impact on inhibitor MIC upon over-expression of tryptophan synthase subunits. The MICs of compounds **1**, **2** and **3** were analyzed with *M. bovis* BCG over-expressing the following pVV16 constructs: empty, *trpA*, *trpA-*D64E, *trpB*, *trpB-*N185S, *trpA-E*. The position of each strain on the agar plate is detailed. The control of 0 μM compound is shown, and the growth of the strains were tested at 2× and 8× MIC of each compound.

The enzymes involved in *de novo* tryptophan biosynthesis are encoded on the *trp* operon (5′-3′): *trpE*, *Rv1610*, *trpC*, *trpB*, *trpA*. To confirm that compounds **1**, **2** and **3**, target biological components of the tryptophan biosynthesis, and more specifically tryptophan synthase, the MIC of *M. bovis* BCG strains over-expressing constituents of the *trp* operon in the presence of the inhibitors was evaluated (Fig. 4). The mycobacterial over-expression plasmid, pVV16, was used to generate constructs containing the *trp* operon (*trpE-A*), *trpB*, *trpB*-N185S, *trpA* and *trpA*-D64E. The mutation *trpB*-N185S was selected because it was present in a number of sequenced spontaneous resistant mutants (Table 2). For the generation of a mutant TrpA, the mutation D64E was chosen. D64 is a conserved residue involved in the communication between the α- and β-subunits, as well as playing a role in the opening and closing of the substrate tunnel between the subunits^14^. Over-expression of the individual WT subunits did not alter the MIC of the compounds. The over-expression of one subunit would not increase the copy number of tryptophan synthase complexes within the cell. With no impact on the MICs, this indicates that the inhibitors do not bind into the active site of either subunit and are targeting the whole complex. This coincides with SNPs observed in both *trpA* and *trpB*. It is noteworthy that the individual subunits may not exist in a conformation conducive to inhibitor binding, which could change upon complex formation. Nevertheless, the MICs were increased with the over-expression of the individual mutant subunits; the tryptophan synthase complex would exist as mixed mutant and WT copies, and although the total copy number of tryptophan synthase complexes would remain the same, the mutation enabled the mutant complexes to function in the presence of the compounds. Over-expression of the *trp* operon also resulted in an increase in the MIC. All components of the tryptophan biosynthetic pathway would have been over-expressed, including both subunits of the tryptophan synthase. Therefore, the total copy number of tryptophan synthase would have been increased, allowing the cell to survive at higher concentrations of inhibitors. These results confirm that tryptophan synthase is the target of the anti-tubercular compounds **1**, **2** and **3**. The involvement of AnsP2 in the resistance phenotype is currently under investigation and is yet to be determined.

### Mechanism of inhibition evaluation

Once a target has been confirmed, establishing the mode of inhibition by a compound is important in its progression through the drug discovery pipeline. Given that the three compounds target the tryptophan synthase complex, and not the individual subunits, there are a number of ways the compound can exert its effect: preventing complex formation, disrupting the complex after it has formed, blocking the conformational changes required for activity, or obstructing the substrate channel between the α- and β-subunits. In order to probe these possibilities, the specific activity of TrpB was evaluated, which catalyses the synthesis of l-tryptophan from indole (generated by the activity of the α-subunit) and l-serine, using the cofactor pyridoxal phosphate (PLP). Recombinant histidine-tagged *Mtb* TrpA, *Mtb* TrpB and *Mtb* TrpB-F188L were over-expressed in *E. coli* and purified by immobilised metal affinity chromatography and anion exchange. The purified enzymes in different conditions (specifically using the substrates to monitor TrpB activity) were evaluated by thin layer chromatography (TLC), native polyacrylamide gel electrophoresis (PAGE) and by gel filtration high-pressure liquid chromatography (GF-HPLC).

**Figure 5:**
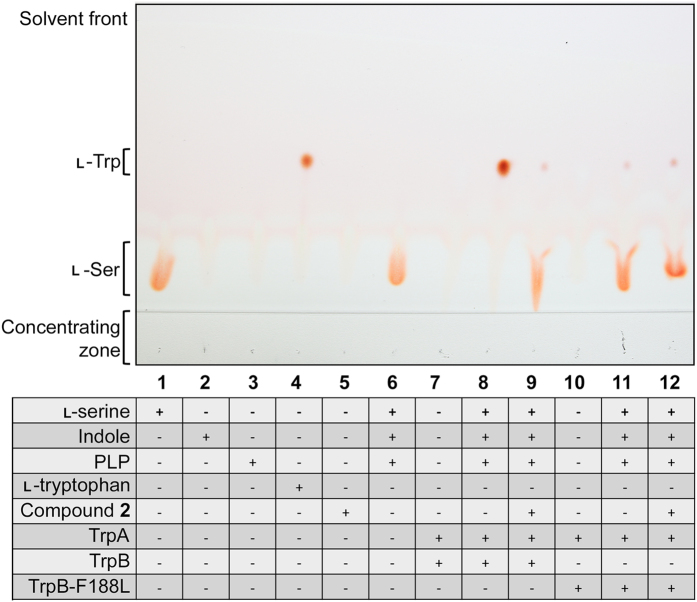
TLC analysis of tryptophan synthase activity and compound **2** inhibition. TrpA and TrpB or TrpB-F188L were incubated with substrates and the tryptophan produced was analyzed by TLC. In the presence of compound **2**, the TrpB activity of the complex was clearly inhibited. However, the TrpB-F188L activity in the presence or absence of inhibitor was comparable. The individual reaction components were analyzed as controls. The bands representing l-serine and l-tryptophan are shown, and the remaining reaction components had no concernable bands in the solvent system and stain used.

TLC was used to analyze the activity of TrpB and TrpB-F188L in combination with compound **2** (Fig. 5). This method enables the production of l-tryptophan to be visualised, confirming TrpB activity. It has been reported that TrpB can function in the absence of TrpA, but the presence of the α-subunit enhances activity^15^. We were unable to establish TrpB activity in the absence of TrpA (Supplementary Fig. S1). Therefore, both enzymes were incubated in equimolar quantities. Figure 5 shows that TrpB actively synthesised l-tryptophan from l-serine and indole (lane 8), and that the synthesis was markedly decreased in the presence of inhibitor (lane 9). TrpB-F188L was also shown to produce l-tryptophan (lane 11), although comparatively less to the WT enzyme (lane 8). However, in the presence of compound **2**, TrpB-F188L generated relatively the same amount of tryptophan (lane 12) compared to when in the absence of inhibitor (lane 11). Although this method of tryptophan production is not quantifiable, it is apparent that the mutation imparts a fitness cost, but concurrently alleviates the impact of the inhibitor.

**Figure 6:**
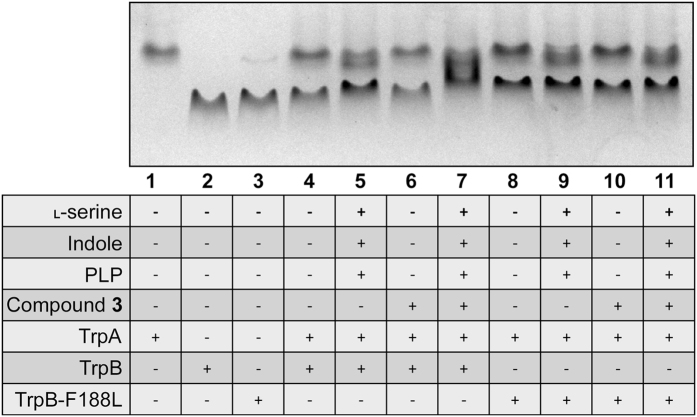
Native PAGE analysis of tryptophan synthase. The relative positions of 3.6 μM TrpA, TrpB and TrpB-F188L on a native PAGE were analyzed, individually, in combination, and in the presence of substrates and compound **3** as detailed. TrpA and TrpB (or TrpB-F188L) did not form a complex, which was also not observable in the presence of inhibitor. In the presence of substrates, the complex formed, which was enhanced with inhibitor in the WT but not the mutant complexes.

The native PAGE analysis of the tryptophan synthase is shown in Fig. 6. The individual subunits of TrpA, TrpB and TrpB-F188L were analyzed individually (lanes 1-3) and in equimolar combinations (TrpA with either TrpB or TrpB-F188L) (lane 4 and 8). Discrete bands for each subunit were observed in each case. In the presence of the inhibitor (compound **3**) (lanes 6 and 10), this result does not change, indicating that the compound cannot induce complex formation. In the presence of the TrpB substrates, however, an additional band was observed, suggesting the formation of WT and mutant complexes (lanes 5 and 9). Furthermore, additional bands were observed in the presence of substrates and inhibitor for the WT complex (lane 7), but not for the mutant (lane 11). Tryptophan synthase is a dynamic enzyme, traversing through a number of conformational states in the synthesis of l-tryptophan^16^. In the presence of the inhibitor, complex formation appeared to be enhanced. It is possible that the inhibitor locks the enzyme in one conformation, preventing structural fluctuations required for activity. These results warranted further investigation by GF-HPLC.

**Figure 7:**
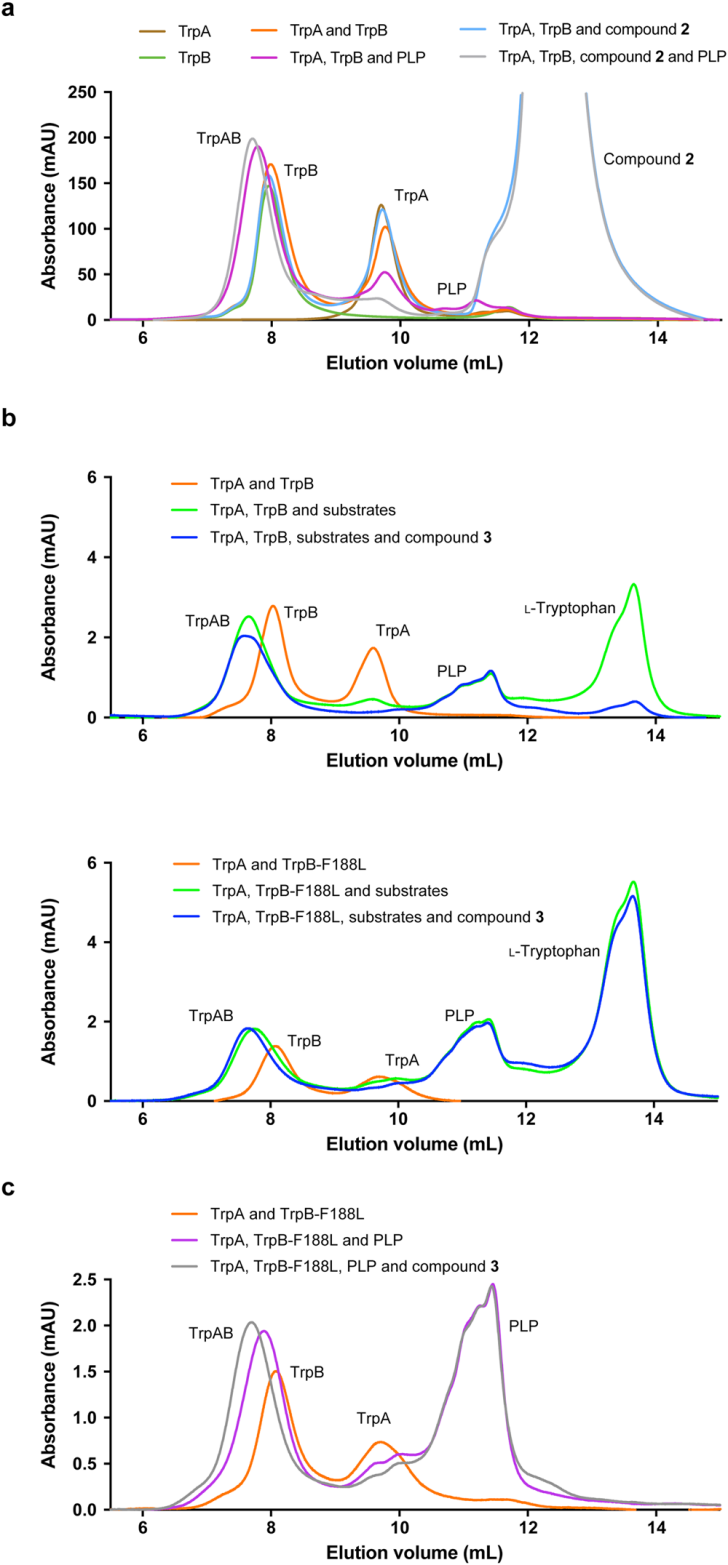
Insights into inhibitor mode of action using gel filtration of tryptophan synthase. Gel filtration was used to analyze the TrpA and TrpB subunits and conditions required for complex formation to give insights into the impact of the TrpB-F188L mutation and the mode of action of the inhibitors. (**a**) Complex formation requirements of tryptophan synthase. TrpA and TrpB eluted as individual peaks in the presence or absence of compound **2**. PLP induced complex formation, which was enhanced in the presence of the inhibitor. Samples were incubated for 1 h and analyzed with absorbance at 210 nm. (**b**) l-Tryptophan synthesis by TrpB and TrpB-F188L in the presence and absence of compound **3**. Compound **3** inhibited l-tryptophan synthesis by TrpB, but not TrpB-F188L. Samples were incubated for 1 h and 2 h for TrpB and TrpB-F188L containing reactions respectively and analyzed with absorbance at 280 nm (to observe l-tryptophan). (**c**) Mutant tryptophan synthase (TrpB-F188L) complex formation is driven by compound **3**. As observed with WT TrpB, PLP induced complex formation and this was further enhanced by the inhibitor. Samples were incubated for 1 h and analyzed with absorbance at 280 nm. The peaks are labeled accordingly. The samples are colored as follows: TrpA, brown; TrpB/TrpB-F188L, green; TrpA and TrpB/TrpB-F188L, orange; TrpA, TrpB/TrpB-F188L and inhibitor (light blue); TrpA, TrpB/TrpB-F188L and PLP, purple; TrpA, TrpB/TrpB-F188L, PLP and inhibitor, grey; TrpA, TrpB/TrpB-F188L, PLP, indole and l-serine, light green; TrpA, TrpB/TrpB-F188L, PLP, indole, l-serine and inhibitor, dark blue.

The GF-HPLC results corroborate the observations made from the native PAGE. TrpA and TrpB each eluted as a single peak from the gel filtration column, where TrpB exists as a dimer in solution. The subunits were analyzed in equimolar concentrations, individually, together and in combination with compound **2** (Fig. 7a). Under these conditions, there was no observable peak shift illustrating complex formation. To probe the precise requirements of complex formation as observed using native PAGE, the cofactor and substrates were incubated with TrpA and TrpB. PLP alone was found to be capable of driving complex formation, as signified by peak shifts: the TrpA peak reduced and the TrpB peak shifted to a higher elution volume, signifying a higher molecular weight and complex formation. Interestingly, upon incubation of the enzymes with PLP and compound **2**, there was further reduction in the TrpA peak and an additional subtle shift to a higher molecular weight of the peak representing the complex. There was no peak shift of TrpB when it was incubated with PLP and inhibitor. These results indicate that the inhibitor does not block complex formation, nor disrupts the complex once it has formed. Intrinsic tryptophan fluorescence binding assays were performed with compounds **2** and **3** (Supplementary Fig. S2) indicating binding of both inhibitors to the PLP-induced tryptophan synthase complex.

l-tryptophan production by the WT TrpB and TrpB-F188L was also visualised using GF-HPLC (Fig. 7b). In the presence of TrpA, substrates and cofactor, WT TrpB is able to synthesise l-tryptophan. However, with the addition of compound **3** (inhibitor does not co-elute with l-tryptophan, unlike compound **2**), there was a significant decrease in the l-tryptophan produced. The same analyzes were performed using TrpB-F188L. It is clear, that even with the inhibitor present, l-tryptophan was produced. To determine whether the F188L mutation prevents the binding of the inhibitor to the complex, enabling l-tryptophan synthesis, TrpA and TrpB-F188L were incubated with PLP (to generate the complex) and compound **3** (Fig. 7c). Surprisingly, the complex peak shifted to a lower elution volume as observed with the WT subunits. These results demonstrate that the inhibitor was still able to bind to the mutant complex.

### Structural insights into the location of resistance conferring mutations

**Figure 8:**
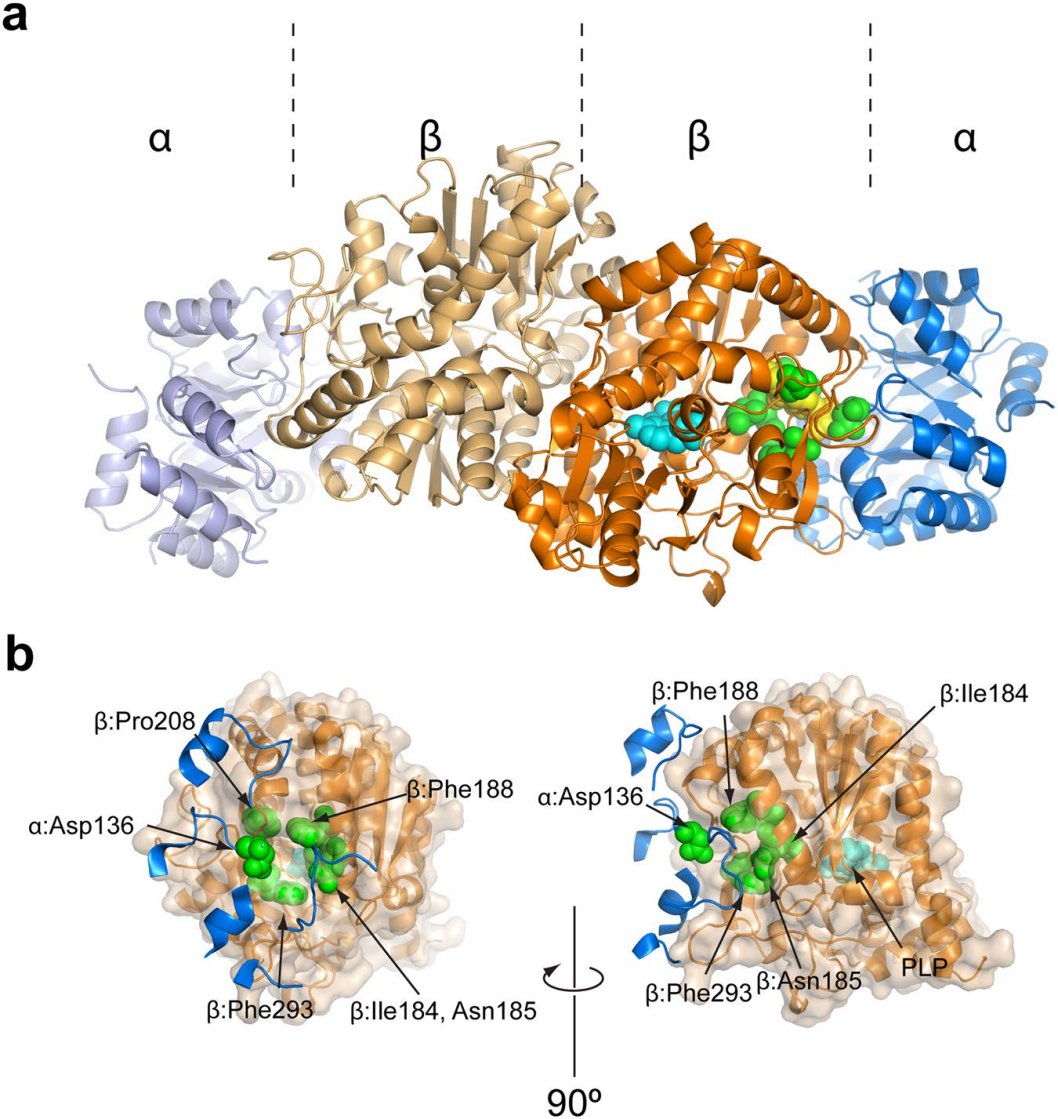
Location of resistance-conferring mutations in tryptophan synthase. (**a**) Ribbon diagram of *Mtb* tryptophan synthase. Residues appearing in resistance-conferring mutations are shown in spheres (green) as is the PLP co-factor (cyan). The inhibitor-binding site is indicated by spheres in yellow. (**b**) Close-up view of the cluster of resistance sites. Left and right panels are linked by a 90° rotation about the vertical direction. The β-subunit is shown as a ribbon (orange) with a transparent molecular surface, while parts of the α-subunit backbone (blue) are shown for residues in contact with the β-subunit surface. Resistance sites (green) and the PLP co-factor (cyan) are indicated.

We attempted to determine the structure of *Mtb* tryptophan synthase complex bound to compound **2** by X-ray crystallography, yielding a low resolution (4.0 Å) structure (Fig. 8a). The 4.0 Å X-ray diffraction data set could be phased by molecular replacement (MR, see Methods for details). Despite the limited resolution, confidence in the MR solution is justified by several criteria: a) high Z-scores (Phaser: Z > 10); b) a complete crystallographic lattice (i.e. all subunits make packing interactions with either crystallographic or non-crystallographically related neighbors); c) clearly discernible difference in density for the PLP co-factor in all β-subunits plus additional density for the N-terminal helix of the TrpA subunit, which was absent in the search model. In total, 6 copies of the α_2_β_2_ heterotetrameric complex were placed in the orthorhombic unit cell (space group *F*222), to yield a crystallographic lattice in which 4 copies of the α_2_β_2_ complex form a ring-like structure, and rings assemble to form pseudo-trigonal nodes in the 3-dimensional lattice (Supplementary Fig. S3).

We mapped the sites of spontaneous resistance-conferring mutations onto the (partially refined) MR solution (Fig. 8). The resistance-conferring mutations cluster at the interface between the α- and β-subunits (Fig. 8a). While mutations on the β-subunit dominate, the mutation α:D136N is situated on a surface element of the α-subunit that covers the substrate tunnel between the α- and β-subunits (Fig. 8b). This structural evidence is consistent with the size-exclusion chromatography results, indicating that adding inhibitor to the PLP-loaded α_2_β_2_ complex further promotes complex formation between the two subunits. Finally, we observe a well-defined patch of difference density at contour levels of 2.5 σ in a site surrounded by the resistance mutations (Supplementary Fig. S3). This density patch is clearly visible in 9 of the 12 α-β interfaces. Shape and extent of this density patch are entirely compatible with the molecular structure of the compound **2**, and indicate a consistent orientation of the inhibitor (Supplementary Fig. S3). The sulfoxide group faces the hydrophilic α-β interface, packing against α:Asp136, one of the resistance sites. The fluorine substituted phenol ring packs against a hydrophobic environment, formed by Phe202, Phe211 and the resistance site Pro208. The central indole moiety forms stacking interactions with Phe188, also a resistance site. While the structural detail revealed by a 4 Å map is evidently limited, the location of the difference density, its consistent appearance in 9 of 12 β-subunits at contour levels distinctly above noise, and the immediate proximity to the sites of resistance-conferring mutations, including α:D136, clearly support the interpretation of this density patch as the inhibitor. Thus, the crystallographic evidence in conjunction with the location of resistance conferring mutations strongly suggest that the inhibitor binds at the interface between the two subunits and may inhibit diffusion of the indole ring from the active site of the α-subunit to that of the β-subunit.

## Discussion

The increasing prevalence of drug-resistant strains of *Mtb* has made more urgent the need for novel inhibitor scaffolds targeting unexploited biosynthetic pathways. Phenotypic HTS strategies have driven the identification of small molecule inhibitors of *Mtb* ^7, 8^ and equally important, inhibitors from these screens are directing the discovery of novel targets, whilst also renewing the potential of recognized, unexploited ones^10–12, 17–19^. In this work, we have identified two new chemical scaffolds with anti-tubercular activities. Through a number of *in vivo* and *in vitro* experiments, we have confirmed tryptophan synthase as the target of the two structurally distinct inhibitor series.

Tryptophan synthase, composed of a heterotetramer of TrpA and TrpB subunits, catalyzes the final step in tryptophan biosynthesis. The α-subunit cleaves indole-3-glycerol phosphate to indole and glycerophosphate, the former diffusing to the active site of the β-subunit where it is covalently bound to l-serine forming l-tryptophan in a PLP-dependent oxidation/reduction reaction. We have shown that the tryptophan synthase complex, rather than its individual subunits, is the target of compounds **1**, **2** and **3**. This is supported by evidence from the sequencing of spontaneous resistant mutants raised against the compounds, which revealed mutations in both *trpA* and *trpB*.

Assignment of the tryptophan synthase complex as the drug target is also supported by over-expression data in *M. bovis* BCG. Over-expression of the individual WT subunits did not alter the MIC of compounds **1**, **2** and **3**. In contrast, in the strains where the tryptophan synthase complex was over-expressed (*trpA-E*), an increase in resistance was observed. Furthermore, there was an increase in resistance of strains that over-expressed mutant copies of the individual subunits. Finally, supplementing minimal media with l-tryptophan rescued the growth inhibition of *M. bovis* BCG caused by compounds **1**, **2** and **3**. Taken together, the resistance, overexpression and supplementation data clearly support the notion of the tryptophan synthase complex as the validated target of the three compounds.

Biochemical studies were used to probe the mechanism of action of inhibitors, and establish how the specific mutations imparted resistance. First, TLC (Fig. 5) was used to establish TrpB activity (in the presence of TrpA) (lane 8). The activity was markedly reduced in the presence of the inhibitor compound **2** (lane 9). Conversely, the activity of TrpB-F188L (lane 11) was significantly less than the WT counterpart, but it retained comparable levels of activity in the presence of the inhibitor (lane 12). Therefore, the resistance conferring mutation, whilst alleviating the impact of the inhibitor, imparts a fitness cost on enzyme activity. In a whole cell setting, the activity of the mutant enzyme must be sufficient because it allows cell survival in high concentrations of drug.

Native PAGE (Fig. 6) and gel filtration (Fig. 7) were used to analyze the effect of the inhibitor on complex formation. Native PAGE demonstrated that complex formation occurred in the presence of all substrates (lanes 5 and 9), and this could not be induced by the inhibitor alone (lanes 6 and 10). These results were corroborated by gel filtration. Native PAGE also demonstrated that inhibitor appeared to drive complex formation (lane 7), potentially trapping the complex in one conformational state. This was not observed with TrpB-F188L (lane 11), and led us to investigate further using gel filtration. As anticipated from the native PAGE, the inhibitor was shown to drive and not disrupt complex formation of the subunits, and that PLP specifically was a requirement for complexation. Surprisingly, this result was emulated by TrpB-F188L. Therefore, the inhibitors were still able to bind to the mutant complex, and in the presence of indole and l-serine, l-tryptophan was produced. The α- and β-subunits are proposed to switch between catalytically inactive (open) and active (closed) conformations^20^. Communication between the two sites enables the activities to be coordinated, preventing the escape of indole and enabling this product of the α-subunit to be channeled through a hydrophobic tunnel to the β-subunit. It is therefore plausible that whilst the inhibitor is still able to bind to the mutant complex, the F188L mutation allows a degree of flexibility, enabling substrate channeling and communication between the subunits.

Inter-subunit communications of tryptophan synthase subunits are under allosteric control, and mediated by a region named the COMM domain^21^. Changes at either active site result in a disruption of interactions with the COMM domain, causing molecular movements to transmit the signal^22^. The cooperativity between the subunits ensures that indole is only synthesized at the α-site following the reaction of l-serine at the β-site. The well-studied *Salmonella typhimurium* tryptophan synthase crystal structure reveals a 25 Å hydrophobic tunnel connecting the catalytic sites of the α- and β-subunits, which is modulated by a molecular gate formed by the residues β-Tyr279 and β-Phe280^21^. The COMM domain controls the opening of the gate to allow the passage of indole through the tunnel for reaction at the β-site and l-tryptophan formation. To establish the roles of the resistance conferring mutations, we have aligned the *Mtb* TrpA and TrpB protein sequences (former residue), with those of the *S. typhimurium* TrpA and TrpB (latter residue): α-D136/D130; β-I184/C170; β-N185/N171; β-F188/L174; β-P208/P194; β-F293/Y279. These residues locate to the α-β inter-subunit interface, also confirmed by the X-ray crystal structure, and are involved in the global motion of the α-subunit relative to the β-subunit, particularly residues β-174–179^21^. There are a number of closely interacting inter- and intra-subunit pairs including α-D130-βPro18, α-D56-N171 and β-N171-βY279^14, 21^. The β-N171 residue of the latter pair is involved in the reorientation of the β-Y279 gate. β-C170 is located on the wall of the tunnel, where mutations to bulky side chains restrict tunnel access and impair α-β subunit communication^22^. It is therefore plausible that the inhibitors bind at the interface, blocking substrate channeling or inter-subunit communication. The resistance conferring mutations could introduce an additional level of flexibility, alleviating any rigidity imposed by the inhibitor, or in the case of the β-F293C (equivalent to β-Y279), the mutation to a less bulky side chain might prevent access restriction of the tunnel caused directly by the inhibitor or through the blocking of the conformational communications.

Tryptophan biosynthesis represents an attractive drug target. The genes encoding the tryptophan synthase subunits are essential, as are the remaining genes in the tryptophan biosynthetic pathway. In fact, mycobacteria have a number of essential genes to synthesise all amino acids *de novo*. Inhibition of the synthesis of a single or multiple amino acids would impose auxotrophism in *Mtb*, necessitating an external amino acid source for cell survival. In the case of tryptophan biosynthesis, the bioavailability of l-tryptophan is limited; in response to *Mtb* infection, macrophages in the granuloma express the indoleamine 2,3-dioxygenases (IDO), metabolizing l-tryptophan to l-kynurenine, thus enhancing the impact of inhibitors^23, 24^. Therefore, inhibition of l-tryptophan biosynthesis in the setting of IDO activity is an innovative strategy for TB chemotherapy. In this work we have validated the therapeutic relevance of tryptophan synthase *in vivo*: in a mouse model with an incipient immune response (IDO activation and tryptophan catabolism are limited), compound **4** was efficacious. Better results are expected in clinical environments in which the host response has tryptophan starvation mechanisms fully active.

*Mtb* amino acid biosynthesis has been largely unexplored from a drug target perspective. The fundamental requirement of amino acids for viability makes the inhibition of these processes suitable targets to pursue. Importantly, the clinical use of inhibitors of amino acid biosynthesis would have a minimal effect on the normal gut flora because it is rich in amino acids that can protect against compound toxicity^25^. Additionally, there is a dietary requirement of a number of amino acids: histidine, isoleucine, leucine, methionine, phenylalanine, threonine, tryptophan and valine. Targeting the biosynthetic pathways against these amino acids, which are likely to lack human orthologues, would reduce the toxic potential of inhibitors. These factors further encourage our exploration into tryptophan biosynthesis as a target of anti-tubercular compounds.

## Materials and Methods

We can confirm that the human biological samples were sourced ethically and their research use was according to the terms of the informed consent, relevant guidelines and regulations at GSK Tres Cantos Medicines Development Campus. We can confirm that all animal studies were ethically reviewed and carried out in accordance with European Directive 2010/63/EU and the GSK Policy on the Care, Welfare and Treatment of Animals. All experimental procedures were performed at GSK Tres Cantos Medicines Development Campus under the supervision and approval from GSK’s local Ethics Committee and corresponding authority (Comunidad Autónoma de Madrid, CAM).

### Compound synthesis

Compounds **2**, **3**, and the racemate of compound **1** were purchased from commercial suppliers. All reagents are also available from commercial vendors.

### (3*R*,4*R*)-3-(4-(2-chlorophenyl)piperazin-1-yl)-4-hydroxytetrahydrothiophene 1,1-dioxide (compound 1)

100 mg of racemic 3-(4-(2-chlorophenyl)piperazin-1-yl)-4-hydroxytetrahydrothiophene 1,1-dioxide were separated by chiral HPLC (flow: 18 mL/min; eluent: hexane/EtOH (90:10); column: Chiralpak IC, 250 × 20 mm). The desired (3*R*,4*R*) eluted at 41.3 min while the other enantiomer (3*S*,4*S*) eluted at 35.1 min. The absolute configuration was determined by VCD.

### (3*R*,4*R*)-3-(4-(2-chlorophenyl)piperidin-1-yl)-4-hydroxytetrahydrothiophene 1,1-dioxide (compound 4)

6-Oxa-3-thiabicyclo[3.1.0]hexane 3,3-dioxide (8.7 g, 64 mmol) was added to 4-(2-chlorophenyl)piperidine hydrochloride (7.5 g, 32 mmol) and Et_3_N (9.9 mL, 71 mmol) in toluene (150 mL). The mixture was stirred at 120 °C for 16 h and then concentrated. Water was added. Extraction (3x) with CH_2_Cl_2_/MeOH (95:5), drying (Na_2_SO_4_) and purification in silica gel using mixtures of CH_2_Cl_2_ and MeOH as eluent followed by crystallization from isopropanol afforded 2.01 g of racemic 3-(4-(2-chlorophenyl)piperidin-1-yl)-4-hydroxytetrahydrothiophene 1,1-dioxide. 50 mg of racemic 3-(4-(2-chlorophenyl)piperidin-1-yl)-4-hydroxytetrahydrothiophene 1,1-dioxide were separated by chiral HPLC (flow: 25 mL/min; eluent: heptane/EtOH with 0.2% of isopropylamine (70:30); column: Chiralpak IA, 300 × 250 mm). The desired (3*R*,4*R*) eluted at 36 min while the other enantiomer (3*S*,4*S*) eluted at 23 min. The absolute configuration was assigned based on their biological activity. 1 H NMR (400 MHz, DMSO-d6) δ ppm: 7.41 (dd, 1 H, *J* = 1.2 and 8.1), 7.38 (dd, 1 H, *J* = 1.7 and 7.8), 7.32 (dt, 1 H, *J* = 1.2 and 7.3), 7.23 (dt, 1 H, *J* = 1.7 and 7.8), 5.63 (d, 1 H, *J* = 5.4), 4.47–4.40 (m, 1 H), 3.51–3.46 (m, 1 H), 3.41–3.36 (m, 1 H), 3.30–3.24 (m, 1 H), 3.21–3.16 (m, 1 H), 3.06–2.88 (m, 4 H), 2.39–2.29 (m, 2 H), 1.77–1.74 (m, 2 H), 1.69–1.57 (m, 2 H). [ES^+^ MS] m/z 330 (M + H)+.

### (3*R*,4*R*)-3-(4-(2-chloro-4-fluorophenyl)piperidin-1-yl)-4-hydroxytetrahydrothiophene 1,1-dioxide (compound 5)

The racemate of compound **5** was obtained by reaction of 6-oxa-3-thiabicyclo[3.1.0]hexane 3,3-dioxide and 4-(2-chloro-4-fluorophenyl)piperidine following the same procedure described for compound **4**. 50 mg of racemic 3-(4-(2-chlorophenyl)piperidin-1-yl)-4-hydroxytetrahydrothiophene 1,1-dioxide were separated by chiral HPLC (flow: 40 mL/min; eluent: heptane/EtOH (80:20); column: Chiralcel OJ-H, 300 × 250 mm). The desired (3*R*,4*R*) eluted at 15.2 min while the other enantiomer (3*S*,4*S*) eluted at 19.0 min. The absolute configuration was assigned based on their biological activity.

1 H NMR (400 MHz, DMSO-d6) δ ppm: 7.44–7.38 (m, 2 H), 7.20 (dt, 1 H, *J* = 2.7 and 8.3), 5.63 (d, 1 H, *J* = 5.4), 4.47–4.40 (m, 1 H), 3.51–3.46 (m, 1 H), 3.41–3.36 (m, 1 H), 3.30–3.24 (m, 1 H), 3.21–3.16 (m, 1 H), 3.06–2.84 (m, 4 H), 2.39–2.29 (m, 2 H), 1.75–1.72 (m, 2 H), 1.68–1.59 (m, 2 H). [ES^+^ MS] m/z 348 (M + H)+.

### HepG2 cytotoxicity

HepG2 cells were cultured using Eagle’s minumim essential medium (MEM) supplemented with 10% heat-inactivated fetal bovine serum (FBS), 1% non-essential amino acid (NEAA) and 1% penicillin/streptomycin. Prior to addition of the cell suspension, 250 nL of test compounds per well were pre-dispensed in TC-treated black clear-bottomed 384-well plates (Greiner) with an Echo 555 instrument. After that, 25 μL of HepG2 (ATCC HB-8065) cells (~3000 cells/well) grown to confluency in Eagle’s MEM supplemented with 10% heat-inactivated FBS, 1% NEAA and 1% penicillin/streptomycin were added to each well with the reagent dispenser. Plates were allowed to incubate at 37 °C with 20% O_2_ and 5% CO_2_ for 48 h.

After the incubation period (48 h), the plates were equilibrated to room temperature before proceeding to develop the luminescent signal. ATP levels measured with CellTiter Glo kit (Promega) were used as cell viability read-out. 25 μL of CellTiter Glo substrate dissolved in the buffer was added to each well. Plates were incubated at room temperature for 10 min for stabilization of luminescence signal and read on View Lux with excitation and emission filters of 613 and 655 nm, respectively.

### Hydrophobicity assay (ChromlogD)

10 mL of 10 mM DMSO stock solutions were diluted to 750 mL with octanol saturated phosphate buffer pH 7.4 and 160 mL buffer saturated octanol in a 96-well deep well block. Blocks were sealed and inverted for 3 sets of 50 inversions, then centrifuged at 300 g for 20 min. Both phases were then quantified using generic gradient UV-HPLC.

### Microsomal fraction stability

Pooled mouse and human liver microsomes were purchased from Xenotech. Microsomes (final protein concentration 0.5 mg/mL, 5 mM MgCl_2_) and test compound (final substrate concentration 0.5 µM; final DMSO concentration 0.5%) in 0.1 M phosphate buffer pH 7.4 were pre-incubated at 37 °C prior to the addition of NADPH (final concentration 1 mM) to initiate the reaction. The final incubation volume was 600 µL. Control samples were included for each compound tested where 0.1 M phosphate buffer pH 7.4 was added instead of NADPH (minus NADPH). Midazolan was included as control in every experiment. Each compound was incubated for 30 min and samples (90 µL) were taken at 0, 5, 10, 20 and 30 min. The minus NADPH control was sampled at 0 and 30 min only. The reactions were stopped by the addition of 200 µL of acetonitrile:methanol (3:1) containing an internal standard, followed by centrifugation at 3700 rpm for 15 min at 4 °C to precipitate the protein. Quantitative analysis was performed using specific LC-MS/MS conditions. Data analysis: from a plot of ln peak area ratio (compound peak area/internal standard peak area) against time, the gradient of the line was determined. Subsequently, half-life and intrinsic clearance were calculated using the equations below:

$$\mathrm{Elimination\; rate\; constant}(k)=(-{\rm{gradient}})$$

$${\rm{Half}}\,{\mathrm{life}(t}_{1/2})(\min )=\frac{0.693}{{\rm{k}}}$$

$$\mathrm{Intrinsic\; Clearance}(\mathrm{CLint})(\mathrm{mL}/\,\min \,/\mathrm{g\; protein})=\frac{{\rm{V}}\times 0.693}{{{\rm{t}}}_{1/2}}$$

where V = Incubation volume mL/g microsomal protein.

### Chemiluminescent nitrogen detection (CLND) solubility

5 mL of 10 mM DMSO stock solution was diluted to 100 mL with phosphate-buffered saline, pH 7.4, equilibrated for 1 h at room temperature, and filtered through Millipore Multiscreen HTS-PCF filter plates (MSSL BPC). The eluent was quantified by suitably calibrated flow injection CLND.

### MIC evaluation and generation of spontaneous resistant mutants

*M. bovis* BCG strain Pasteur and derivatives were cultured in static liquid or solid medium at 37 °C, 5% CO_2_. Liquid medium: Middlebrook 7H9 (Difco), 0.05% (v/v) Tween-80, 10% (v/v) Middlebrook ADC and 0.25% (v/v) glycerol. Solid medium: Middlebrook 7H11 agar (Difco) with 10% (v/v) Middlebrook OADC and 0.5% (v/v) glycerol. Where applicable, 25 μg/mL kanamycin was added to the media to select for mycobacterial plasmids. The MIC was determined by plating 10^4^, 10^3^, 10^2^, and 10^1^ cells from a mid-log culture of *M. bovis* BCG on solid medium containing increasing concentrations of inhibitor in a dose response. The MIC was the lowest concentration of compound that caused complete inhibition of bacterial growth. *M. bovis* BCG spontaneous resistant mutants were generated on solid media at 5×, 10×, and 20× MIC. Resistant isolates were confirmed by first culturing in liquid media (in the absence of compound) before retesting on solid media containing 5× MIC of each compound. The genomic DNA was extracted and purified according to standard procedures. Photographs of agar plates were taken with a Canon PowerShot G16 and Biorad Gel Doc XR+ using Image Lab software. Images were processed using Adobe Illustrator 5.0 software.

### Sequencing of *M. bovis* BCG spontaneous resistant mutants

*M. bovis* BCG WT and resistant mutant strains were characterised by WGS. Briefly, the genomic DNA was prepared for sequencing by the generation of DNA libraries (Nextera DNA Sample Preparation Kit, Illumina), which were purified (Agencourt AMPure XP beads, Beckman Coulter Genomics), quantified (Quant-iT PicoGreen dsDNA kit, Life Technologies) and the fragment sizes were evaluated (2100 Bioanalyzer with a High Sensitivity DNA chip, Agilent Technologies). The DNA libraries were sequenced on a MiSeq Benchtop Sequencer (MiSeq Reagent Kit v2, 300 cycles). The genome sequence of *M. bovis* BCG Pasteur 1173P2 (accession: NC_008769.1) was used as a reference to align reads.

### Generation of plasmid expression constructs

The mycobacterial and *Escherichia coli* over-expression constructs were generated in the plasmids pVV16 and pET28a respectively, both of which encode kanamycin selection. For constructs generated in pVV16, the stop codon was not included in the antisense primer, enabling the C-terminal vector-encoded histidine tag to be exploited. Conversely, primers designed for the generation of pET28a constructs, the stop codon was included, where a vector encoded N-terminal histidine tag is employed. Initially, primers were designed to clone *trpA*, *trpB* (and the mutant equivalents) and the *trp* operon (pVV16 only) into the multiple cloning sites of pVV16 and pET28a, exploiting the restriction sites *Nde*I and *Hin*dIII. The primers are listed in Table 3. Following the manufacturer’s instructions, the genes were amplified by PCR from *M. bovis* BCG (WT or mutant) genomic DNA using Q5 DNA polymerase (New England Biolabs). Following purification (QIAquick PCR Purification Kit, Qiagen), the PCR products and the vectors were digested with the restriction enzymes specified (FastDigest, Thermo Fisher Scientific), and the digest products were purified via gel extraction from a 1% agarose gel (QIAquick Gel Extraction Kit, Qiagen). Digest products were ligated using T4 DNA ligase (New England Biolabs) and transformed into *E. coli* TOP10 cells. The constructs in positive clones were verified by DNA sequencing (Eurofins Genomics).

**Table 3:** Primers used in the generation of over-expression constructs.

Primer	Plasmid	Sequence (5′-3′)
*trpA* sense	pVV16, pET28a	GATCGATC**CATATG**GTGGCGGTGGAACAGAGCGAAG
*trpA* antisense	pVV16	GATCGATC**AAGCTT**TGCGGACATCCCTAGTCGTACCC
	pET28a	GATCGATC**AAGCTT**TTATGCGGACATCCCTAGTCGTACCC
*trpB* sense	pVV16, pET28a	GATCGATC**CATATG**AGTGCTGCCATCGCCGAACCG
*trpB* antisense	pVV16	GATCGATC**AAGCTT**GTCGTTGCCCAGCAAGCCAAACC
	pET28a	GATCGATC**AAGCTT**TTAGTCGTTGCCCAGCAAGCCAAACC
*trpE-A* (operon) sense	pVV16	GATCGATC**CATATG**CACGCCGACCTCGCAGCCAC
*trpE-A* (operon) antisense	pVV16	GATCGATC**AAGCTT**TGCGGACATCCCTAGTCGTACCC

The gene-specific sense and antisense primers are displayed and the cloning vector specified. The primers can be used to amplify the WT or mutant copies of the genes, depending on the genomic DNA used. Restriction sites (*Nde*I and *Hin*dIII) are highlighted in bold type.

### Electroporation of mycobacterial expression constructs

Electrocompetent *M. bovis* BCG cells were prepared by pelleting and subsequently washing a mid-log culture with decreasing volumes of ice-cold 10% (v/v) glycerol. The cells were incubated on ice with 1 μg plasmid DNA, before being transferred to a 0.1 cm electrode gap electroporation cuvette. A single pulse of 1.8 kV was applied, and the cells were recovered by incubation overnight in liquid media, before positive clones were selected on solid medium containing the appropriate antibiotic.

### l-Tryptophan supplementation experiments

The MIC of compound **1** against *M. bovis* BCG was analyzed on solid minimal medium in the presence of 0.01% l-tryptophan. The minimal media (pH7.5) contained 11 mM KH_2_PO_4_, 0.8 mM MgSO_4_, 4.6 μM ferric ammonium citrate, 25 mM Na_2_HPO_4_, 19 mM NH_4_Cl, 0.14 mM CaCl_2_, 14.5 mM NaCl using 0.1% glycerol as a carbon source and 1.5% agar. The MIC was evaluated as previously described.

The impact of *M. bovis* BCG viability in the presence of 1×, 5× and 10× compound **2** or **3** was analyzed in liquid minimal media with and without 0.01% l-tryptophan. Cells were cultured to OD_600 nm_ 0.6–0.8 and diluted to 1 × 10^6^ colony-forming units (CFU)/mL (OD_600 nm_ 1.0 = 2.5 × 10^8^ CFU/mL) in minimal media with and without 0.01% (v/v) l-tryptophan. 1 μL compound (100% DMSO) (using stocks of 5 mM, 25 mM and 50 mM compound **2** or 0.7 mM, 3.5 mM and 7 mM compound **3** (1×, 5× and 10× MIC)) and appropriate controls (1 μL DMSO or 1 μL 1 mM INH) were aliquoted into a 96-well plate (flat, black bottom, polystyrene) in duplicate. 99 μL 1 × 10^6^ CFU/mL ( +/−l-tryptophan) were added to each well and then the plate was incubated at 37 °C, 5% CO_2_ for 7 days. 30 μL 0.02% (w/v) resazurin and 12.5 μL 20% (v/v) Tween 80 was added and the plate was incubated in the same conditions for 24 h. Fluorescence was measured by λ excitation at 530 nm and λ emission at 590 nm using a POLARstar Omega plate reader.

### Expression and purification of recombinant TrpA and TrpB

Recombinant TrpA and TrpB were over-expressed in BL21 (DE3) *E. coli* cells from the pET28a constructs. TrpA over-expression was optimised using the Studier method of autoinduction^26^. Cells were incubated at 37 °C, 180 rpm, until OD_600 nm_ 0.2 was reached. The temperature was reduced to 20 °C and the cells were cultured overnight before harvesting. Recombinant TrpB was over-expressed by 0.5 mM IPTG induction of a mid-log culture grown in terrific broth at 37 °C, 180 rpm. Following induction, the cells were cultured overnight at 16 °C before harvesting.

Recombinant TrpA and TrpB were purified using identical methods. Cell pellets were resuspended in buffer (50 mM sodium phosphate, pH 7.5, 0.5 M NaCl and 25 mM imidazole) containing DNAse and Complete protease inhibitor cocktail tablets (Roche). Cells were sonicated, 30 s on, 30 s off, for 12 cycles. Insoluble material was pelleted at 18,000 rpm. The clarified lysate was applied to a 1 mL, pre-equilibrated (in buffer) nickel-charged IMAC column. A step gradient of 25, 50, 100, 150, 200, 300 and 1000 mM imidazole (in buffer) was used to wash the column and elute the recombinant proteins. TrpA eluted at 100 mM imidazole, whereas the cleanest fraction of TrpB eluted at 150 mM imidazole. The proteins were immediately buffer-exchanged into 50 mM Tris, pH 7.5, 10% (v/v) glycerol. Anion exchange was performed, where the proteins eluted from a 1 mL Q HP anion exchange column over an increasing NaCl gradient. Purified proteins were stored at −20 °C.

### TLC

In order to visualise amino acids by TLC, 5 μg is needed (10 μL loaded). Therefore, each reaction was performed in 100 μL with substrates (indole and l-serine) and control (l-tryptophan) at 0.5 μg/μL. Each reaction was performed in 100 mM Tris, pH 7.5, 150 mM NaCl. TrpA and TrpB/TrpB-F188L were present in equimolar quantities (10 μM each). PLP was added to 100 μM. Where applicable, compound **2** was added to 100 μM, and replaced by an equivalent volume of DMSO. The final DMSO concentration of reactions was 1% (v/v). The reactions were incubated for 1 h at room temperature. Once loaded, the TLC (silica gel 60) was developed in butanol:acetic acid:H_2_O (12:3:5 (v/v/v)). Ninhydrin was used to detect the amino acids. TLCs were imaged using a Canon EOS 5D Mark III and processed using Adobe Illustrator 5.0 software.

### Native PAGE

Samples were prepared for native PAGE with the following concentrations in combinations as stated (buffer, 50 mM Tris pH 7.5, 150 mM NaCl; total volume, 10 μL): 3.6 μM TrpA, 3.6 μM TrpB, 20 μM compound **3**, 1 mM indole, 1 mM PLP, 100 mM serine. Samples were diluted in an equal volume of sample buffer (62.5 mM Tris-HCl, pH 6.8, 40% (w/v) glycerol, 0.01% (w/v) bromophenol blue) and resolved in 25 mM Tris, 192 mM glycine, using a precast gel (Biorad). Images were taken using a Biorad Gel Doc XR+ with Image Lab software, and processed using Adobe Illustrator 5.0 software.

### GF-HPLC

HPLC using a gel filtration column (TSKgel G3000SW_XL_, 50 mM Tris pH 7.5, 150 mM NaCl) was used to analyze TrpA and TrpB, measuring absorbance at 210 nm or 280 nm. Samples were prepared in 50 μL with the following components (in combinations as stated): 20 μM TrpA, 20 μM TrpB/TrpB-F188L, 100 μM PLP, 100 μM indole, 100 μM serine, 100 μM compound **2**/**3**. The reaction buffer was 50 mM Tris pH 7.5, 150 mM NaCl. Samples were incubated at room temperature for 1–2 h (as specified). GF-HPLC was performed on a Thermo Scientific Ultimate 3000, and data was recorded on Chromeleon software. Images were generated using GraphPad Prism 5.

### Crystallisation conditions

Purified recombinant TrpA and TrpB were incubated together in equimolar concentrations (8.64 μM) with 86.4 μM PLP, 400 μM indole and 40 mM l-serine in a final volume of 6 mL. After incubation at room temperature for 1 h, compound **2** was added to 86.4 μM and the reaction was incubated for a further 1 h before concentrating to 120 μL (equivalent to 10 mg/mL TrpA). Crystallisation conditions were screened using a number of sparse matrix screens (Molecular Dimensions) with 300 nL sitting drops (150 nL drops of protein solution and screen) dispensed by a liquid handling robot. Crystals formed in 0.1 M HEPES, pH 6.0, 50% polypropylene glycol 400, 5% DMSO at 18 °C. X-ray diffraction data were recorded at the Diamond Light Source.

### X-ray crystallographic structure determination

X-ray data to 4.0 Å resolution were recorded on beamline I04 at the Diamond Light Source (Supplementary Table S1), using a Pilatus 6 M area detector (DECTRIS). The X-ray diffraction images were integrated and scaled using the software XDS, XSCALE^27^, and AIMLESS of the CCP4 package^28^. The structure was solved by molecular replacement, using the PHASER software^29, 30^ and the heterotetrameric TrpAB complex from *Pyrococcus furiosus* as a search model (pdb entry 5E0K^31^, 33% identity for TrpA, 58% for TrpB). The search model was stripped of side chains that are not conserved between the target and the *P. furiosus* structure. Given the large unit cell, the Matthews coefficient analysis did not yield a definitive guide to the number copies present in the asymmetric unit. Nevertheless, placing copies of the heterotetrameric complex consecutively and selecting the correct solution based on the Z-score (18.5 to 21.3) of the translation function allowed to unequivocally determine the position of 6 copies of the αββα complex, leading to a crystallographically plausible 3D lattice (Supplementary Fig. S3). Later on, coordinates of the refined structure of the TrpAB complex of *M. tuberculosis* became available in the PDB (entry 5TCG, 2.4 Å resolution^32^). This coordinate set was stripped of water molecules and co-factors, mapped onto the molecular solution, and used to correct the structural model. The resulting density map consistently showed density for the PLP co-factor, the coordinates of which were excluded from the search model. Density was also visible for N-terminal helix of the TrpA subunit, which is absent in the *P. furiosus* ortholog. The additional density, together with the high translation function Z-scores, gave confidence that the correct solution had been found. A partial refinement of the molecular replacement solution led to an R_free_ of 36.9%, refining coordinates, and modelling temperature factors through TLS refinement and a uniform B-factor for each protein chain. The difference density map in Supplementary Fig. S3 was calculated based on protein coordinates, prior to incorporation of the co-factor or ligands in the model. The model of the inhibitor compound **2** was placed manually into the unbiased difference density and adjusted using real space refinement^33^, but not included in the crystallographic refinement of the model.

### Mice

Specific Pathogen-free 6–8-week-old female C57BL/6 J mice (18–20 g) were obtained from Envigo (Barcelona, Spain). The experiments were performed at AAALAC-accredited GlaxoSmithKline Laboratory Animal Science animal facilities in Tres Cantos (Madrid, Spain). The mice were kept in air-filtered isolators with twenty air changes per hour. Room temperature and relative humidity were 22 ± 2 °C and 55 ± 10%, respectively. The mice were accommodated in groups of up to five individuals in Tecniplast® type IV cages with autoclaved dust free corncob bedding (Rettenmaier Iberica, S. L., Barcelona, Spain). The mice were maintained under a 12 h light/dark period. Filtered tap water and γ-irradiated pelleted diet from Envigo (Barcelona, Spain) were provided *ad libitum*.

### *In vivo* efficacy assessment

The experimental design has been previously described^34^. In brief, mice were intratracheally infected with 100,000 CFU/mouse (*Mtb* H37Rv). Products were administered for 8 consecutive days starting one day after infection. Lungs were harvested 24 h after the last administration. All lung lobes were aseptically removed 24 h after the last administration, homogenized and frozen. Homogenates were plated in 10% OADC-7H11 medium for 14 days at 37 °C. Homogenates from compound treated mice were incubated for 18 days at 37 °C in plates supplemented with 0.4% (wt, vol) activated charcoal (Sigma Aldrich) to prevent the effect of product carryover. Moxifloxacin (Sequoia Research Products Ltd) was prepared as solution in 20% Captisol(R)/water.

Blood samples (aliquots of 15 μL) were taken from the infected mice by the lateral tail vein at the following time points: compound **4** (oral route): 0.5, 2, 6, 24, 48, 120, 168 and 192 h; compound **5** (subcutaneous route): 0.17, 1, 6, 24, 48, 120, 168 and 192 h. LC-MS/MS was used as the analytical method for the determination of compound concentration in blood. Plots of blood levels were generated with GraphPad Prism 6 (GraphPad Software, Inc).

### Statistical analysis

Graphic data were prepared with GraphPad Prism software and the results were analyzed as stated in the text.

## Electronic supplementary material

Supplementary Information
