# Supplementary material for: Inhibiting mycobacterial tryptophan synthase by targeting the inter-subunit interface
Source: Sci Rep. 2017 Aug 25;7:9430. doi: 10.1038/s41598-017-09642-y (PMC5573416; doi:10.1038/s41598-017-09642-y)
Supplement: Supplementary file 1 — Supplementary Information [file 41598_2017_9642_MOESM1_ESM.pdf]

SUPPLEMENTARY INFORMATION

Inhibiting mycobacterial tryptophan synthase by targeting the inter-subunit interface

Katherine A. Abrahams[1], Jonathan A. G. Cox[2], Klaus Fütterer[1], Joaquín Rullas[3], Fátima Ortega-Muro[3], Nicholas J. Loman[1], Patrick J. Moynihan[1], Esther Pérez-Herrán[3], Elena Jiménez[3], Jorge Esquivias[3], David Barros[3], Lluís Ballell[3], Carlos Alemparte[3*], Gurdyal S. Besra[1*]

[1]Institute of Microbiology and Infection, School of Biosciences, University of Birmingham, Edgbaston, Birmingham B15 2TT, UK, [2]School of Life and Health Sciences, Aston University, Aston Triangle, Birmingham, B4 7ET, [3]Tres Cantos Medicines Development Campus, GlaxoSmithKline, Severo Ochoa 2, 28760 Tres Cantos, Madrid, Spain

[*]E-mail for correspondence: g.besra@bham.ac.uk (TEL: +00 44 121 415 8125; FAX +00 44 121 414 5925), carlos.g.alemparte@gsk.com (TEL: +00 34 638 498 619; FAX +00 34 91 807 0550)

Table of contents:

Supplementary Methods p2

Supplementary Table p3

Supplementary Figures p4

Supplementary Methods

Intrinsic tryptophan fluorescence inhibitor binding assays

Fluorescence binding assays of the tryptophan synthase complex (3 μM TrpA, 3 μM TrpB, 30 μM PLP) with compounds **1**, **2** and **3** were performed in 50 mM Tris pH7.5, 150 mM NaCl, 10% glycerol, at 25°C with sequential additions of compound (in 100% (v/v) DMSO). Fluorescence was monitored in a 500 μl crystal cuvette using a Hitachi F7000 Fluorescence Spectrophotometer at an excitation wavelength of 280 nm and emission wavelength of 300-400 nm (excitation and emission slit width of 5 nm). DMSO concentrations were <1.1% (v/v) in each assay. Changes of fluorescence intensities due to volume expansion and non-specific binding of DMSO were accounted for. Data were recorded on Hitachi FL Solutions 4.6 software and analysed using Prism 5 (GraphPad).

**X-ray diffraction data**	
**Data collection**	
Beamline	Diamond I02
Wavelength (Å)	0.97949
Space group	*F*222
Unit cell parameters (Å)	426.1, 432.1, 434.0
Number of $\alpha_2\beta_2$ complexes per asymmetric unit	6
Resolution limits (Å)	49.8 - 4.0
Resolution shell (Å)	4.07 - 4.0
Number of observations	586405
Number unique reflections	160634
Rmerge (%)	13.0 (67.4)
Mean I / σ(I)	6.4 (1.2)
Mn(I) half-set correlation CC(1/2)	0.994 (0.539)
Completeness (%)	96.3 (75.5)
Multiplicity	3.7 (2.4)
**Refinement**	
Resolution range	49.8 – 4.0
Unique reflections	156,611
R_{cryst} (%), R_{free} (%)	35.8, 36.8
No. of non-hydrogen atoms	57,878
RMSD bonds (Å), angles (º)	0.021, 1.7
Average B-factor (Å^2)	126.44
RMSD B-factor (Å^2)	1.2
Ramachandran Plot	
Favoured region (%)	94.2%
Disallowed region (%)	0.2%

Supplementary Table S1: Data collection and refinement statistics. Values in parentheses are for the highest-resolution shell. Collection based on a single crystal.

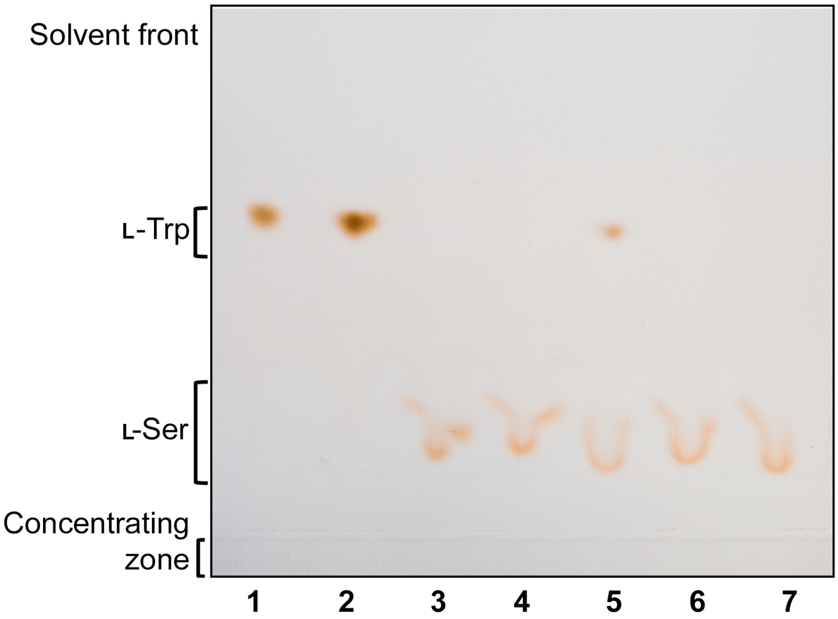

	1	2	3	4	5	6	7
L-serine	-	+	+	+	+	+	+
Indole	-	+	+	+	+	+	+
PLP	-	+	+	+	+	+	+
L-tryptophan	+	-	-	-	-	-	-
Compound **2**	-	-	-	+	-	-	+
TrpA	-	+	-	-	+	-	+
TrpB	-	+	+	+	-	-	-
TrpB-F188L	-	-	-	-	+	+	+

Supplementary Figure S1: TLC analysis of TrpB and TrpB-F188L activity. TrpA and TrpB or TrpB-F188L were incubated together with substrates and the L-tryptophan generated was analyzed by TLC. The relative amounts of L-tryptophan synthesized by the WT and mutant complexes were compared to the activity of TrpB or TrpB-F188L alone. TrpB and TrpB-F188L were seemingly inactive in the absence of TrpA, and the inhibitor had no apparent effect. Therefore, it was not possible to determine whether TrpB activity is directly inhibited by compound **2**, or whether there is a requirement for complex formation.

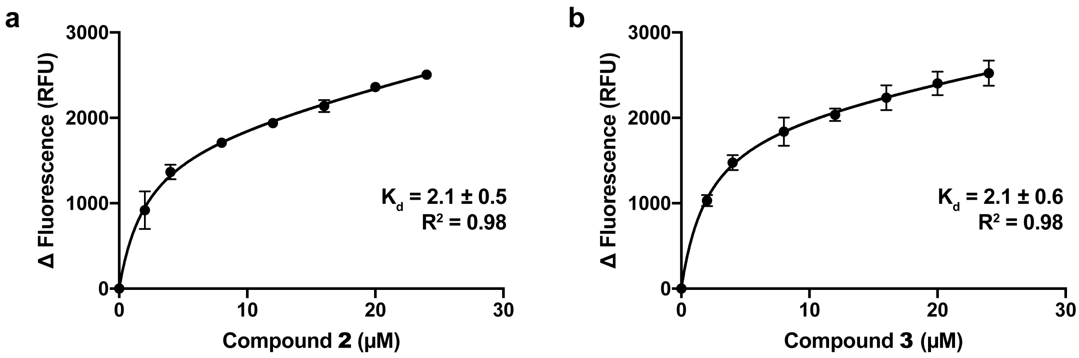

Supplementary Figure S2: Intrinsic tryptophan fluorescence inhibitor binding assays. Intrinsic tryptophan fluorescence binding assays were used to quantify the association of the tryptophan synthase complex with compounds **2** and **3**. Data were fitted using GraphPad Prism and the K_d values (mean ± s.e.m. based on n=3) from non-linear least-squares fitting of a single site binding model and R^2 values are shown. RFU, relative fluorescence units.

a

b

c

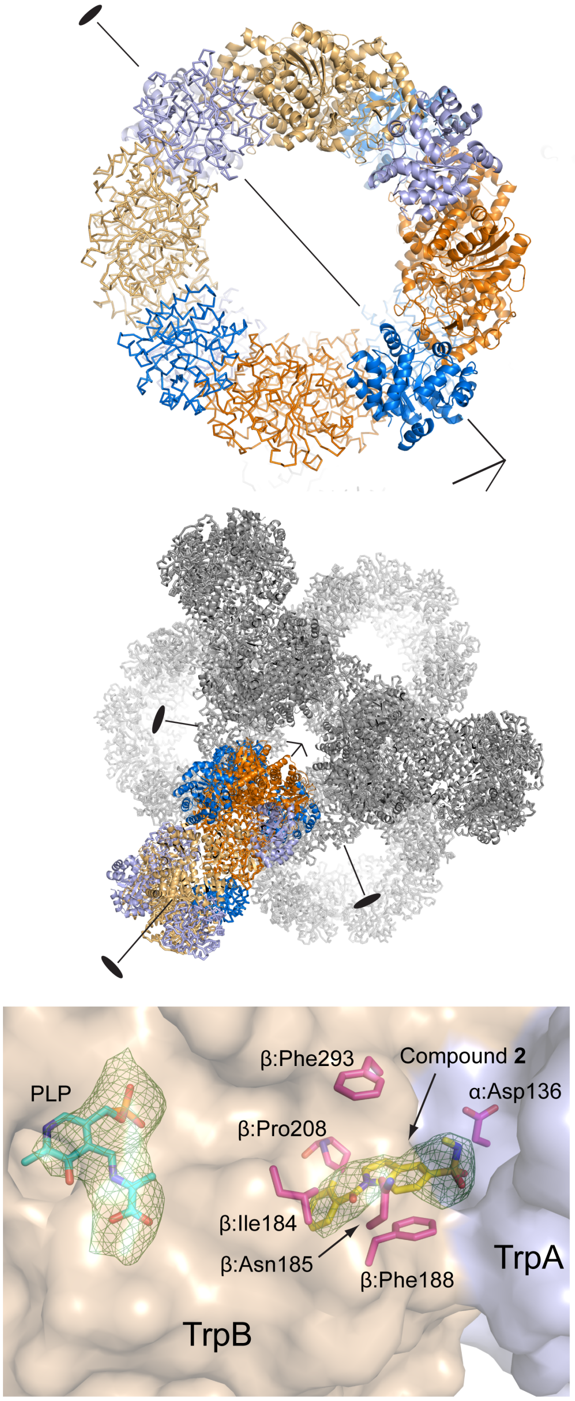

Supplementary Figure S3: Crystal lattice of *Mtb* tryptophan synthase MR solution. a) Ring-like structure formed by 4 copies of $\alpha_2\beta_2$ complex, with α- and β-subunits appearing in hues of blue and orange, respectively. Complexes in the asymmetric unit and symmetry-related copies are drawn as ribbons and Cα-traces, respectively. b) Pseudo-trigonal nodes in the orthorhombic lattice formed by the ring structures depicted in panel *a*. Crystallographic 2-fold rotation axes are indicated by

straight lines and the ellipse symbols. c) Difference density for co-factor PLP and compound **2**. The α- and β-subunits are shown in surfaces rendered in blue and brown, respectively. The sites of resistance-conferring mutations are indicated by sticks in magenta. The difference map, calculated using model phases prior to incorporation of co-factor or inhibitor in the coordinate set, is contoured at 2.5 σ.
